# Cerebral Haemorrhage in a Young Patient With Atypical Werner Syndrome Due to Mutations in *LMNA*

**DOI:** 10.3389/fendo.2018.00433

**Published:** 2018-08-03

**Authors:** Xiao Yanhua, Zhou Suxian

**Affiliations:** ^1^Affiliated Hospital of Guilin Medical College, Guilin, China; ^2^Guilin People's Hospital, Guilin, China

**Keywords:** progeroid, Werner syndrome, *LMNA*, cerebral haemorrhage, gene mutation

## Abstract

**Introduction:** Werner syndrome is a rare genetic disorder; classical Werner syndrome is caused by mutations in the *WRN* gene. However, recent research has shown that *LMNA* gene mutations can also cause premature ageing syndromes such as atypical Werner syndrome (AWS). AWS usually manifests as muscular damage, defects in the cardiac conduction system, lipoatrophy, diabetes, atherosclerosis, and premature ageing.

**Clinical presentation:** A 24-year-old man presented with severe abdominal aortic and peripheral artery disease and cerebral haemorrhage. He was prescribed once-daily 20 mg atorvastatin. Another large cerebral haemorrhage occurred 8 months after discharge. Although he underwent minimally invasive intracranial haematoma surgery, paralysis set in. Molecular studies showed a missense mutation within exon 5 (c.898G>C) that caused amino acid aspartate 300 to be replaced by histidine (p.Asp300His) in the *LMNA* gene. The patient was diagnosed with AWS.

**Conclusions:** Haemorrhagic stroke and progeroid features may be manifestations of *LMNA*-linked AWS. In such cases, the patient's family history and genetic background should be investigated. *WRN* and *LMNA* gene testing of the proband and the immediate family should be considered. This case report provides a deeper understanding of the role of *LMNA* mutations in AWS.

## Introduction

Werner syndrome (WS) is defined as a segmental autosomal recessive progeroid disease that has the main manifestations of premature ageing and early death. Classical WS is caused by mutations in the *WRN* gene. The central region of the WRN protein contains the RECQ helicase domain. RECQ is a DNA helicase that plays an important role in DNA replication, repair, and stability (1, 2). Recent research has shown that *LMNA* gene mutations can cause premature ageing syndromes such as Hutchinson-Gilford progeria syndrome and atypical Werner syndrome (AWS) (2). *LMNA* mutations also cause conditions known as laminopathies; these include Emery-Dreifuss muscular dystrophy, expansionary disease, and lipoatrophy.

WS is a rare recessive inherited condition. Individuals with WS often present with an aged appearance, loss of body hair, cataracts in both eyes, lipoatrophy, skin pigmentation, a beaked nose, diabetes, hyperlipaemia, and atherosclerosis (3). The incidence rate of tumours in WS patients is up to 60 times that of their unaffected peers, with thyroid cancers occurring at the highest rate.

Previous literature has described the clinical manifestations of WS as being similar to AWS (4, 5); however, recent studies have indicated that AWS is due to mutations in the *LMNA* gene (6). We identified AWS in a 24-year-old man with a variety of vascular lesions and progeroid features; genetic testing revealed a c.898G>C mutation in *LMNA* that resulted in p.Asp300His.

## Clinical case

**Figure 1:**
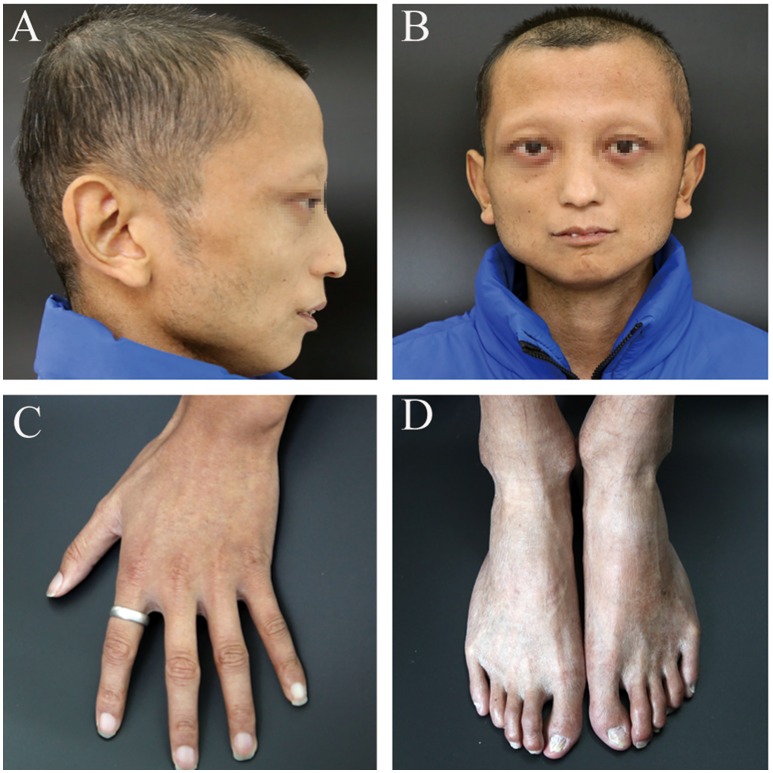
The patient's clinical features. **(A)** beaked nose and sparse scalp. **(B)** “bird” face and sparse eyebrows, exophthalmos. **(C,D)** lipoatrophy and skin pigmentation. Informed consent was obtained from the patient and his parents for all images referenced above to appear in Frontiers.

**Figure 2:**
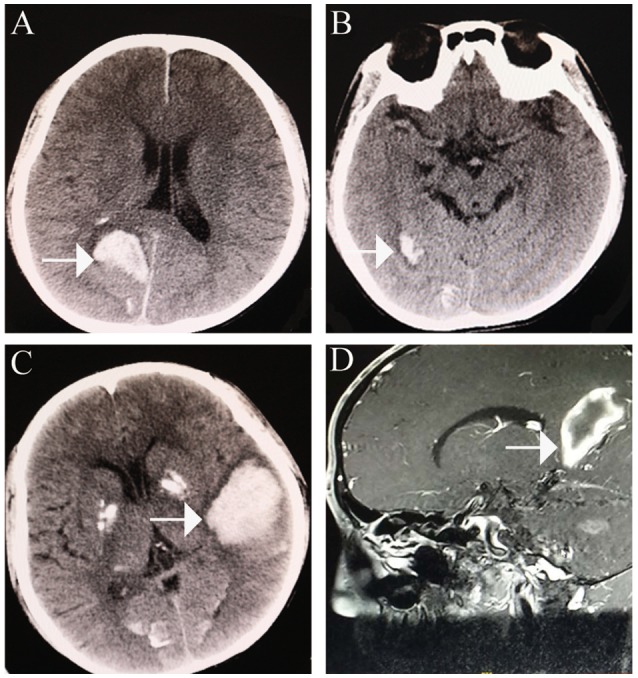
Cerebral haemorrhage. **(A)** cerebral haemorrhage was noted in the right occipital lobe. **(B)** further cerebral haemorrhage in the right occipital lobe. **(C,D)** large area of cerebral haemorrhage in the left medial temporoparietal area.

A 24-year-old man (height: 169 cm, weight: 44 kg, blood pressure: 168/75 mmHg) presented with paroxysmal disturbance of consciousness accompanied by muscle spasms. He was diagnosed with cerebral haemorrhage. He had experienced two previous cerebral haemorrhages between the ages of 23 and 24 years. He was an only child, and his parents did not have any history of haemorrhage or abnormal skin appearance or other medical histories. He was referred to the endocrinology department with multiple intracranial calcifications, acute intracerebral haemorrhage, and several other unusual features. His features were as follows: beaked nose (Figure 1A), “bird” face, light, and sparse scalp and body hair, no obvious armpit hair or eyebrows, exophthalmos (Figure 1B), hoarse voice, lipoatrophy, skin pigmentation (Figures 1C,D), severe abdominal aortic, and peripheral artery disease, cerebral haemorrhage (Figures 2A,B), and erectile dysfunction.

**Figure 3:**
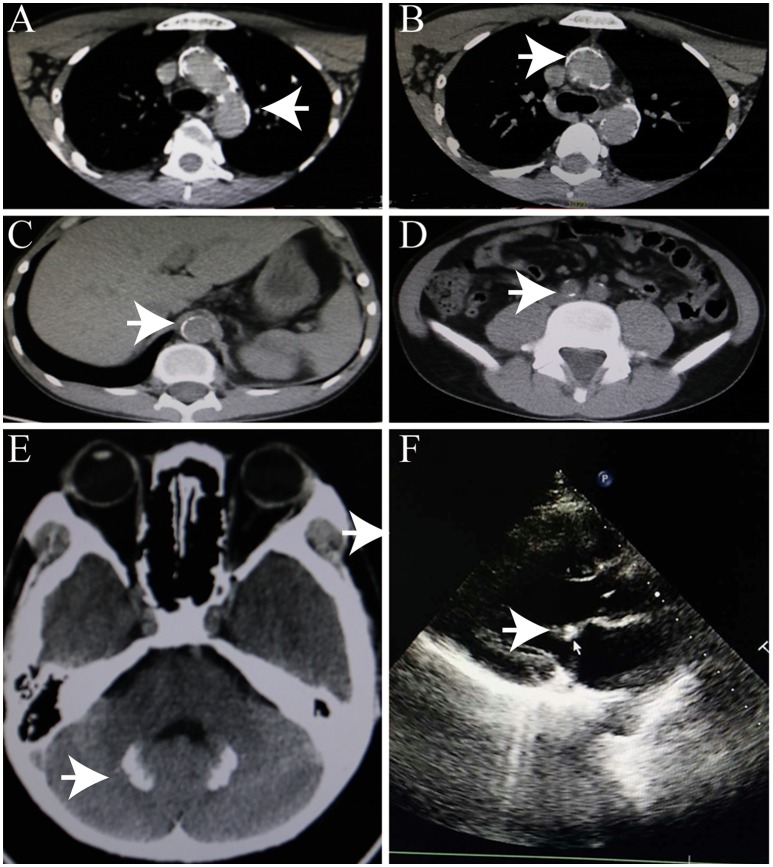
Heterotopic calcification. **(A–C)** Arterial calcification. **(D)** Calcification of head. **(E)** Intracranial calcification. **(F)** Mitral calcification.

An initial brain computed tomography (CT) scan showed that the right occipital lobe was haemorrhagic with approximately 1.5 ml (Figure 2B). CT angiography revealed plaque formation in, and vascular calcification of, the aortic arch, bilateral subclavian artery, brachiocephalic trunk, proximal internal carotid artery, aorta abdominalis, and arteria iliaca communis (Figures 3A–D). Intracranial calcification was also revealed on CT (Figure 3E). Vascular ultrasonography showed atherosclerosis and plaque formation in the intracranial vessels and bilateral carotid and posterior tibial arteries. Doppler ultrasonography showed mitral calcification (Figure 3F). Bone density scans revealed osteopenia (T level−1.8SD); plain skull x-ray imaging also showed decreased bone density. The patient's blood count results were as follows: white blood cells, 15.77 × 10∧9/L; neutrophils, 73.9%;triglycerides (TG) 1.76 mmol/L; cholesterol (CHOL), 6.24 mmol/L; low density lipoprotein cholesterol (LDLc), 5.61 mmol/L; high-sensitivity C-reactive protein (hsCRP), 87.26 mg/L; myocardial enzyme lactate dehydrogenase (LDH), 267.12 U/L; creatine kinase (CK), 899.52 U/L; MB isoenzyme of creatine kinase (CK-MB), 24.34 U/L.

Once-daily atorvastatin (20 mg) was prescribed; however, another large cerebral haemorrhage developed 8 months post-discharge (Figures 2C,D). Although the patient accepted minimally invasive intracranial haematoma surgery, paralysis persisted.

**Figure 4:**
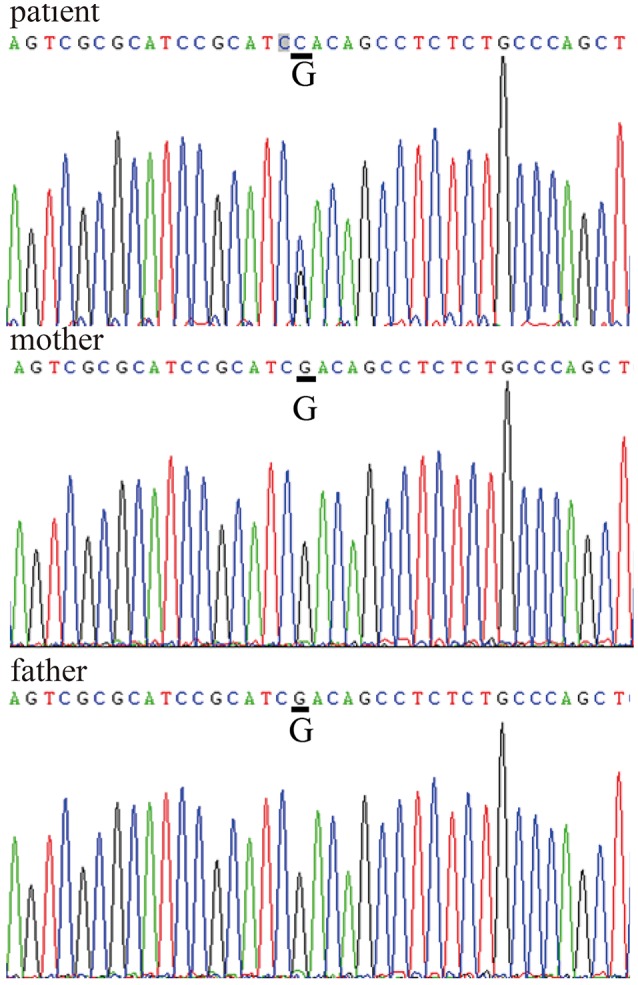
*LMNA* gene sequences *LMNA* mutation within exon 5: c.898G>C, p.Asp300His in the patient's sequence versus his parents' sequences.

After obtaining informed consent from both the patient and his parents, genetic analysis was carried out. We sequenced the *WRN* and *LMNA* (GenBank reference sequence NM_170707.2) coding sequences in the patient and his parents. The results showed a missense mutation within exon 5 of *LMNA* (c.898G>C) that caused a substitution of aspartate 300 by histidine (p.Asp300His). There were no *WRN* mutations (Figure 4). The patient was the only child of his parents. Both parents had a normal appearance, and both carried the wild-type *LMNA* sequence without any symptoms of AWS, cardiovascular disease, or abnormal medical history. This disease is closely linked to mutations in the lamin A/C, or *LMNA*, gene, which confirmed the diagnosis of AWS.

## Discussion

Progeroid syndromes are rare genetic disorders that have accelerated ageing as the primary clinical feature. Other defining characteristics include severe atherosclerosis and vascular disease, both of which are attributed to autosomal *WRN* or *LMNA* mutations (7). *WRN* and *LMNA* are critical for genome replication and stability (8). Previous genetic studies have indicated that classical WS is associated with a *WRN* gene mutation; *WRN* encodes the RECQ DNA helicase family. The incidence rate of WS in Japan is one birth per hundred thousand; in other parts of the world, the rate ranges from one birth per one million to one birth per ten million. The incidence rates are correlated with the levels of consanguineous marriage.

Premature ageing is the main characteristic of WS and AWS. WS is caused by mutations in the *WRN* gene. The central region of the WRN protein contains the RecQ helicase domain, which is an ATP-dependent helicase (9). Recent studies have indicated that AWS is due to mutations in the *LMNA* gene (6). *LMNA* is located on chromosome 1q21-22. It contains 12 exons (4) and encodes the major nuclear proteins lamin A and lamin C. These proteins play an important role in DNA damage replication and maintenance of the double strand (10).

Several recent studies have indicated that both diseases have common pathophysiologic mechanisms and clinical manifestations (11). Between the ages of 23 and 25 years, our patient suffered three cerebral haemorrhages. Through gene sequencing, we ruled out *WRN* mutations as a causative factor. *LMNA* gene sequencing revealed a c.898G>C transition in exon 5, which is a pathogenic mutation that causes AWS with acute ischaemic cerebrovascular disease. The *LMNA* mutation within exon 5 (c.898G>C, p.Asp300His) that we reported here has previously been highlighted as the location of other missense mutations (10). One article reported a mutation in the same location that resulted in a substitution (c.899A>G, p.Asp300Gly, p.D300G) (3). Among three atypical cases, Chen also identified mutations in *LMNA*; these were identified as R133L and L140R (12, 13).

The mechanisms by which mutation of *LMNA* results in AWS are not known. Through alternative splicing, *LMNA* encodes lamin A and C as two protein isoforms (4). Lamins A and C are members of the intermediate filament protein family, which polymerise with B-type vitamins to form a nuclear layer. They constitute the nuclear network under the nucleus of the protein network. Lamins are important in regulating gene transcription and cell mitosis. Mutations in *LMNA* cause multifunctional disorders called laminopathies. *De novo* synthesis of lamin A mutations and heterozygous mutations causes the production of progerin. Progerin is deposited in the nucleus, giving rise to rare atypical progeroid syndromes such as AWS, lipodystrophies, and cardiomyopathy.

Previous studies have described AWS patients as sharing specific clinical features, including prematurely aged appearance, beaked nose, prematurely grey hair, hair loss, hoarse voice, flat feet, lipoatrophy, and diabetes mellitus (4, 5). However, another report found that *LMNA* mutations lead to fibrosis of the dermis, heterotopic calcification, and haemorrhage (5, 14). Generalized multiple atherosclerosis and atheromatous plaques, which relate to diabetes, hyperlipaemia, hypertension, high homocysteine, and hyperuricaemia, were found in our patient (15, 16). Though our patient had experienced three cerebral haemorrhages, the specific mechanism of these haemorrhages was not clear. Daisuke Kinoshita reported that the overexpression of progerin and deletion of p53 leads to the arrest of vascular smooth muscle cell growth. However, DNA-dependent protein kinase (DNA-PK) was activated, suggesting that mutant *LMNA* and progerin activities in WS relate to cardio-cerebral vascular disease (2). In addition, our patient had myocardial lesions; the degree of atherosclerosis may have correlated with the myocardial lesions and haemorrhage (coronary angiography with CT or coronary angiography alone would reveal this). Overexpressing mutant LMNA leads to accelerated telomere attrition in fibroblasts as well as expedited senescence. The mutant protein causes unstable DNA damage that shortens the telomerase, causing TRF2 degradation (6). In AWS, lamin A mutants and wild-type lamin A were able to combine with DNA-PK, through this pathway may result in cardiovascular with atherosclerosis and striking ischaemic events, together with other features of premature ageing (2). Atrioventricular blocks have been reported in a patient with recurrent fainting episodes (17). *LMNA* mutations inhibit vascular smooth muscle cell growth via the DNA damage response pathway, which causes premature tissue ageing and leads to functional defects such as dysfunctional cardiac conduction and myocarditis in muscular dystrophy (2, 18–20). Mutations in *LMNA* also cause lipodystrophy through the activation of MAP kinases; this has been observed in WS (21, 22).

In summary, our patient had AWS caused by a c.898G>C, p.Asp300His mutation in the *LMNA* gene. *LMNA* mutation-related AWS presents with different characteristics, including cerebral haemorrhage, atherosclerosis, beaked nose, and premature hair loss. Since the specific pathogenesis remains unclear, studies exploring the molecular biological mechanisms are necessary.

## Availability of data and materials

Informed consent was obtained from the patient and his parents for all parts of the material referenced above to appear worldwide in published media. The study was conducted in accordance with the declaration of Helsinki.

## Ethics statement

This study was carried out in accordance with the recommendations of Affiliated Hospital of Guilin Medical University, Committee on Biomedical Ethics. The protocol was approved by the Committee on Biomedical Ethics. All subjects gave written informed consent in accordance with the Declaration of Helsinki.

## Author contributions

ZS designed the study and XY is the major contributor in writing the manuscript.

### Conflict of interest statement

The authors declare that the research was conducted in the absence of any commercial or financial relationships that could be construed as a potential conflict of interest.
